# A true theranostic pair – ^44/47^Sc-labeled GRPR antagonist shows great promise for managing prostate and breast cancer

**DOI:** 10.1007/s00259-025-07651-y

**Published:** 2025-11-11

**Authors:** Naveen Kumar, Adrianna Bilinska, Elena Menéndez, Tilman Läppchen, Euy Sung Moon, Malgorzata Zoltowska, Dariusz Pawlak, Izabela Cieszykowska, Renata Mikolajczak, Frank Rösch, Axel Rominger, Eleni Gourni

**Affiliations:** 1 Department of Nuclear Medicine, Inselspital, Bern University Hospital, University of Bern, Bern, Switzerland; 2 Graduate School of Cellular and Biomedical Sciences, University of Bern, Bern, Switzerland; 3 Department of Chemistry - TRIGA site, Johannes Gutenberg University of Mainz, Mainz, Germany; 4 Radioisotope Centre POLATOM, National Centre for Nuclear Research, Otwock, Otwock-Świerk Poland

**Keywords:** GRPR antagonist, ^44/47^Sc, mTOR inhibitor, Everolimus, Fractionated dose

## Abstract

**Purpose:**

This study investigates the theranostic potential of a ^44/47^Sc-labeled antagonist targeting the gastrin-releasing peptide receptor (GRPR) in prostate and breast tumors.

**Methods:**

A statine-based GRPR antagonist (AAZTA^5^-Pip-D-Phe-Gln-Trp-Ala-Val-Gly-His-Sta-Leu-NH_2_: LF1) was radiolabeled with scandium-44/47. Detailed in vitro evaluation was carried out in PC3 and T47D cancer cells. In vivo studies, including blood and organ clearance, plasma protein binding, metabolic stability and SPECT/CT imaging, were performed in PC3- and T47D-mice. To assess its therapeutic efficacy, PC3-mice were treated with [^47^Sc]Sc-LF1 either alone or in combination with everolimus.

**Results:**

[^47^Sc]Sc-LF1 exhibited high binding affinity, low internalization rate (< 10% in PC3 and T47D cells), and favorable pharmacokinetics, including rapid blood clearance and low plasma protein binding. In PC3-mice, it demonstrated high and specific tumor uptake (45.4 ± 3.9 and 4.9 ± 1.6% I.A./g at 4 and 96 h p.i., respectively), while lower GRPR density in T47D-mice led to reduced uptake (6.1 ± 3.9 and 0.7 ± 0.1% I.A./g at 4 and 72 h p.i.). Its pharmacokinetics enabled high-contrast SPECT/CT imaging in both models. Combined treatment with everolimus and [^47^Sc]Sc-LF1 in PC3-mice, significantly inhibited tumor growth compared to monotherapies.

**Conclusion:**

The high tumor uptake in two cancer entities with elevated expression of GRPR and the tumor response (tumor size and survival rate) highlight the significant theranostic potential of [^44^Sc]Sc/[^47^Sc]Sc-LF1 for PET (scandium-44) and SPECT (scandium-47) imaging and radionuclide targeted therapy.

**Supplementary Information:**

The online version contains supplementary material available at 10.1007/s00259-025-07651-y.

## Introduction

The gastrin-releasing peptide receptor (GRPR) is overexpressed in several cancers, including prostate, breast, and lung, making it a valuable target for imaging and targeted radionuclide therapy (TRT) [1, 2].

Early GRPR-targeted peptides were agonists, however, their clinical use was limited by severe gastrointestinal side effects [3, 4]. In contrast, GRPR antagonists have demonstrated superior tumor uptake, longer retention, and reduced off-target effects, key advantages for theranostic applications [5, 6]. These properties could be further improved by labeling with suitable long-lived radionuclides such as ^177^Lu, ^44/47^Sc, or ^90^Y, allowing patients to benefit from late-stage imaging while minimizing radiation exposure to healthy tissues [7–10].

Given the growing significance of TRT, advancements are not only being made in the development of novel targets and delivery vectors, but also in the exploration of suitable radionuclides for clinical application [8, 11]. Lutetium-177 remains a standard radionuclide for TRT and is frequently employed in therapy monitoring via SPECT imaging [11]. However, its emission profile, specifically the 208 keV gamma ray with an 11% abundance, limits spatial resolution and necessitates extended imaging durations [11]. In contrast, scandium-47, a beta-emitting radionuclide with a half-life of 3.35 days, has attracted increasing attention. Owing to its 159 keV gamma emission with a higher abundance (68.3%), scandium-47 presents a promising alternative for both therapeutic use and improved SPECT imaging [11–14]. In addition, its β⁻ particles (Emax = 0.6 MeV) provide selective irradiation of small tumors with minimal damage to surrounding tissue, ideal for treating micro-metastases [11, 13]. Importantly, ^47^Sc is chemically identical to ^44^Sc (t_1/2_=3.97 h), its PET radionuclide counterpart, allowing for pre-therapy PET imaging and post-therapy treatment with the same compound [11, 13, 15, 16]. This matched radionuclide pair supports personalized theranostics with consistent pharmacokinetics and bioavailability [15].

This study focuses on LF1, a GRPR-targeted peptide (H-D-Phe-Gln-Trp-Ala-Val-Gly-His-Sta-Leu-NH₂), conjugated via a Pip spacer to the AAZTA^5^ chelator [17]. We developed a robust radiolabeling protocol for both scandium-44 and scandium-47, achieving high purity and stability. [^47^Sc]Sc-LF1 was evaluated in vitro for receptor binding and internalization, and in vivo for biodistribution, blood clearance, protein binding, and metabolic stability in prostate and breast tumor models. SPECT/CT imaging confirmed tumor targeting. Finally, its therapeutic efficacy was assessed through tumor regression studies using a fractionated dosing scheme as well as combination therapy with everolimus.

Our data demonstrate the translational potential of [^47^Sc]Sc-LF1 as a new class of theranostic agents and support further development toward clinical application in GRPR-expressing malignancies.

## Materials and methods

### Production of scandium-44 and scandium-47

Scandium-44 was eluted from a ^44^Sc/^44^Ti generator located at the Institute of Nuclear Chemistry in Mainz, following established protocols for both elution and post-processing purification [18, 19].

Scandium-47 was produced at Radioisotope Centre POLATOM, National Centre for Nuclear Research (NCBJ), Poland, by neutron irradiation of enriched [^46^Ca]CaCO₃ (10.5% ^46^Ca) (Isolflex, USA) in the Maria research reactor, in the nuclear reaction ^46^Ca(n,γ)^47^Ca→^47^Sc [14]. Targets were encapsulated in quartz ampoules and irradiated at the thermal neutron flux ~ 1 × 10¹⁴ n/cm²/s for 199–262 hours. After irradiation, the targets were dissolved in 3 M HCl. DGA resin (N, N,N’,N’-tetrakis-2-ethylhexyl-diglycolamide based, TrisKem International, France) was used for the selective isolation of [^47^Sc]Sc³⁺ from the bulk calcium matrix and other potential metal impurities. The final product, Sc-47, was eluted as chloride in 0.05 M HCl, a solution suitable for radiolabeling after QC testing comprising the assessment of radionuclidic purity by gamma spectrometry, chemical purity by ICP-OES and radiochemical purity by the ITLC.

### Radiolabeling/quality control/lipophilicity/protein binding studies

LF1 was radiolabeled with scandium-44 and with scandium-47. The quality control of the radiolabeling mixture was performed by radio- TLC and HPLC. The radiochemical stability of [^47^Sc]Sc-LF1 was evaluated for a period of 8 days by radio-TLC analysis. The lipophilicity (LogD_octanol/PBS_) and protein binding were also determined (supplemental data).

### Cell lines

The prostate PC3 and the breast T47D cancer cells have been used for the study. Cultivation conditions, materials and further details are described in the supplemental data.

### Saturation binding studies/internalization/externalization

For receptor saturation studies, PC3 and T47D cells were incubated with 1 to 100 nM of ^nat/47^Sc-LF1. For internalization studies, approximately 2.5 pmol of [^47^Sc]Sc-LF1 was added to PC3 and T47D cells, followed by incubation for 30, 60, 120, 240 and 360 min at 37 °C, 5% CO_2_ (supplemental Data).

### Animal models

Male athymic Balb/C nude mice (age: 6 weeks, weight: 16–20 g) were implanted with prostate PC3 cells (3 × 10^6^/100 µL PBS) into their right shoulder. Female athymic Balb/C nude mice (age: 6 weeks, weight: 16–20 g) were implanted with breast T47D cells (12 × 10^6^ in 100 µL of PBS: Matrigel, 1:1) into their right shoulder. For the development of the breast tumor models, mice were also implanted with home-made silicon-based hormone pellets (1 mg estrogen/pellet) one week before the implantation of the breast cancer cells. The animals were used for biodistribution and SPECT/CT imaging studies, once tumors reached 250–300 mm³.

### Biodistribution studies

A total of 10 pmol of [^47^Sc]Sc-LF1 in NaCl 0.9% (~ 0.02–0.06 MBq/0.1 mL) was injected in the tail vein PC3- and T47D-mice. Animals were terminally anesthetized by intraperitoneal injection of an overdose of pentobarbital sodium solution (150 mg/kg) at 1, 4, 24, 48, 72 and 96 h after the injection of [^47^Sc]Sc-LF1. Selected organs were dissected and weighed, and the radioactivity in the tissue samples was counted in a γ-counter. Biodistribution data are given as per cent of injected activity per gram of tissue (% I.A./g) and expressed as mean values ± SD (*n* = 3–4). To demonstrate the specificity of the binding, blocking studies (*n* = 3) were performed by co-injection of 10 pmol of [^47^Sc]Sc-LF1 (~ 0.02–0.06 MBq/0.1 mL) and 20 nmol of H-D-Phe-Gln-Trp-Ala-Val-Gly-His-Sta-Leu-NH_2_; biodistribution was assessed at 4 h p.i.

### In vivo protein binding/murine plasma metabolic stability studies

Healthy mice (*n* = 2) were injected with 200 pmol of [^47^Sc]Sc-LF1 in saline (~ 6 MBq/0.1 mL) in NaCl 0.9% and sacrificed 5 and 15 min post-injection (p.i.) to determine the percentage of the radiotracer bound to the plasma proteins and the percentage of intact tracer (supplementary data).

### Blood and organ clearance kinetics

Healthy mice (*n* = 2) were injected into the tail vein with 200 pmol of [^47^Sc]Sc-LF1 (~ 6 MBq/0.1 mL in 0.9% NaCl). Blood samples were collected at multiple time points (1–240 min p.i.) and measured using a γ-counter. Blood half-lives were determined with GraphPad Prism using a Two-Phase Decay Model, while tumor, pancreas, and kidney half-lives were calculated from biodistribution data using a One-Phase Decay Model (supplementary data).

### Area under the time-activity curves (AUC)

The areas under the time-activity curves (AUC) in the tumor, blood, pancreas and kidneys were calculated from the non-corrected biodistribution data using GraphPad Prism. The results of AUC are expressed as % I.A./g×h and presented as mean values ± SD (*n* = 3–4).

### Ex vivo autoradiography

Frozen PC3 tumor Sect. (7 μm) from mice injected with 400 pmol (~ 7.5 MBq/100 µL) of [^47^Sc]Sc-LF1 were collected at 1 h p.i. and exposed overnight to an FBCS 810 autoradiography cassette (FisherBiotech). Scanning was performed with a Typhoon 9400 analyzer (50 μm resolution) (Amersham Biosciences/GE Healthcare), and images were analyzed using OptiQuant software.

### Small-animal SPECT/CT imaging

Static SPECT images were obtained upon injection of 400 pmol of [^47^Sc]Sc-LF1 (~ 13–14 MBq/100 µL) in NaCl 0.9% and 200 pmol of [^47^Sc]Sc-LF1 (~ 6–7 MBq/100 µL) in NaCl 0.9% in the tail vein of PC3- and T47D-mice, respectively. Images were acquired at 1, 4, 24, 48, 72 and 96 h p.i., and imaging was performed in spontaneously breathing animals under isoflurane anesthesia (2% isoflurane, 1.5 mL/min oxygen). Blocking studies (*n* = 2) were performed upon co-injection of 200–400 pmol (depends on the tumor model) of [^47^Sc]Sc-LF1 and 20 nmol of the H-D-Phe-Gln-Trp-Ala-Val-Gly-His-Sta-Leu-NH_2_, and the animals were imaged at 4 h p.i. under the same conditions as described above. Further details are given in the supplementary material.

### In vivo monotherapy of PC3 tumor bearing mice using [^47^Sc]Sc-LF1

Tumor regression studies of [^47^Sc]Sc-LF1 were conducted using a fractionated dosing regimen in PC3-mice (Fig. 6A; supplementary material). Once the average tumor volume reached ~ 162 ± 70 mm^3^, mice were divided into four groups (*n* = 4–5/group). Two groups received [^47^Sc]Sc-LF1 on days 0, 2, and 4, and again on days 13, 15, and 17, with total doses of ~ 30 MBq (1200 pmol in total) and ~ 60 MBq (2400 pmol in total), respectively. Control groups received either ^nat^Sc-LF1 (1200 pmol) or PBS, following the same schedule. Tumor size and body weight were monitored three times weekly. Tumor volume was calculated as Width×(Length)^2^ × 0.5. Mice were euthanized when tumors exceeded 1.0 cm^3^, weight loss exceeded 15%, or after 150 days if neither endpoint was reached.

###  In vivo combination therapy with [^47^Sc]Sc-LF1 and everolimus

The therapeutic efficacy of combining [^47^Sc]Sc-LF1 with everolimus was evaluated in PC3-mice (Fig. 7A; supplementary material), using the same tumor size criteria as in monotherapy studies. Three groups of mice (*n* = 4–5 per group) underwent: (a) ***[***^***47***^***Sc]Sc-LF1 monotherapy***: Mice received 400 pmol (10 ± 0.5 MBq) of [^47^Sc]Sc-LF1 on days 0, 2, and 4 of therapy. (b) ***Everolimus monotherapy***: Mice received everolimus (5 mg/kg/day) on days 0, 2, and 4 of the therapy. (c) ***Combination therapy***: Mice were treated with [^47^Sc]Sc-LF1 (400 pmol, 10 ± 0.5 MBq) and everolimus (5 mg/kg/day) on days 0, 2, and 4 of therapy. The total administered radioactivity for groups (a) and (c) was approximately 30 MBq (1200 pmol in total). Monotherapy and combination studies were conducted in parallel, sharing control groups (^nat^Sc-LF1, and PBS), with identical monitoring protocols.

### Morphological assessment

Selected mouse organs (kidney, pancreas and tumor) were dissected from the sacrificed animals and immediately frozen using liquid nitrogen. Frozen tissue samples were sectioned at 7 μm thickness using a cryostat maintained at −20 °C to −25 °C and mounted onto microscope glass slides, followed by staining with hematoxylin and eosin for microscopic examination using standard protocols (supplementary material).

### Statistical analysis

Data are expressed as mean ± standard deviation (mean ± SD). GraphPad Prism version 10.2.0 was used to performed statistical analysis using the log-rank test, with a P value of less than 0.05 considered significant.

## Results

### Radiolabeling/quality control/radiochemical stability of [^44/47^Sc]Sc-LF1

[^47^Sc]Sc-LF1 was generated in molar activities ranging between 5 and 40 GBq/µmol, depending on the planned experiment. Due to the low initial activities of [^44^Sc]Sc^3+^, the molar activity for [^44^Sc]Sc-LF1 was ~ 0.2 GBq/µmol. The radiochemical yield and purity for both tracers were more than 99%, as assessed by radio-TLC and radio-HPLC analysis (Fig. S1). [^47^Sc]Sc-LF1 showed very high radiochemical stability (99%) with no signs of radiochemical degradation or radiolysis up to 96 h after radiolabeling. After 96 h, a tailing was observed in the baseline, and the radiochemical stability was assessed as 92% and decreased to 87% after 192 h (Fig. S2).

### Lipophilicity/protein binding studies

The LogD_octanol/PBS−pH7.4_ values were − 2.63 ± 0.07 and − 2.79 ± 0.02 for [^44^Sc]Sc-LF1 and [^47^Sc]Sc-LF1 respectively. Protein binding was about 10% at 60 min of incubation with human serum at 37 °C for both radioligands.

### Saturation studies

**Fig. 1:**
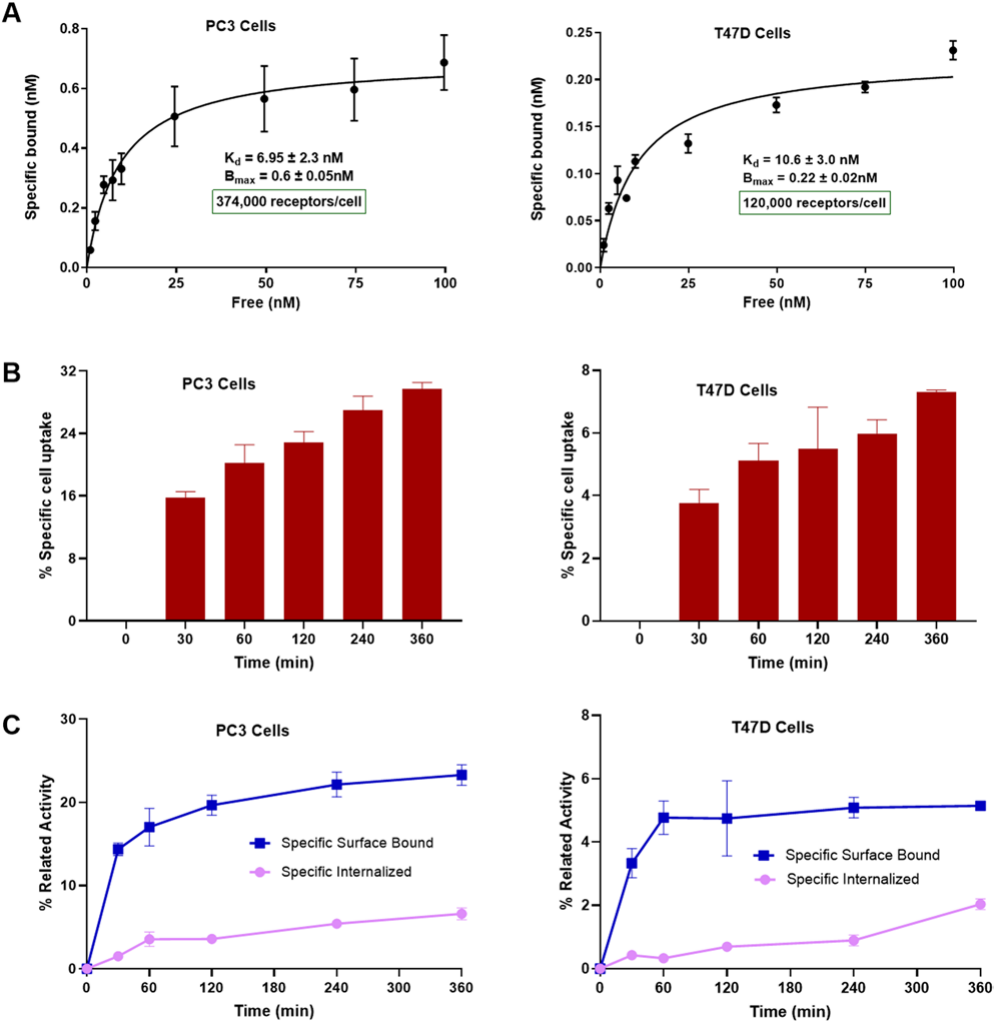
**(A)** Saturation binding study on PC3 and T47D cells, using increasing concentrations of ^nat/47^Sc-LF1 (1 to 100 nM). K_d_ and B_max_ were calculated from nonlinear regression analysis using GraphPad Prism. **(B)** Total specifc cell uptake after the incubation of PC3 and T47D cells with [^47^Sc]Sc-LF1 within 6 h at 37 °C. **(C)** Internalized and specific surface-bound activity after the incubation of PC3 and T47D cells with [^47^Sc]Sc-LF1 within 6 h at 37 °C

Saturation binding studies at 4 °C enabled radioconjugate-receptor binding while preventing endocytosis. ^nat/47^Sc-LF1 exhibited affinity for PC3 and T47D cells, with K_d_ values of 6.9 ± 2.3 nM and 10.6 ± 3.0 nM respectively (Fig. 1A). B_max_ values were 0.6 ± 0.05 nM (~ 3.74 × 10^5^ receptors/cell) for PC3 and 0.22 ± 0.02 nM (~ 1.20 × 10⁵ receptors/cell) for T47D.

### Internalization studies

[^47^Sc]Sc-LF1 was found to be well associated with the PC3 and T47D cells within the incubation time frame (Fig. 1B and C). Continued exposure of the cells to the radioactive ligand resulted in a gradual increase of the total cell-associated uptake from 30 min to 6 h. Total cell-associated uptake at 6 h in PC3 cells was 29.7 ± 0.8% which is fourfold higher as compared to cell-associated uptake in T47D cells (7.23 ± 0.07%). At 6 h, the amount of specifically internalized activity was 6.60 ± 0.17% in PC3 and 2.04 ± 0.17% in T47D cells. Blocking experiments performed with an excess of H-D-Phe-Gln-Trp-Ala-Val-Gly-His-Sta-Leu-NH_2_ (1 µM) showed negligible nonspecific cell binding.

### Biodistribution studies

**Fig. 2:**
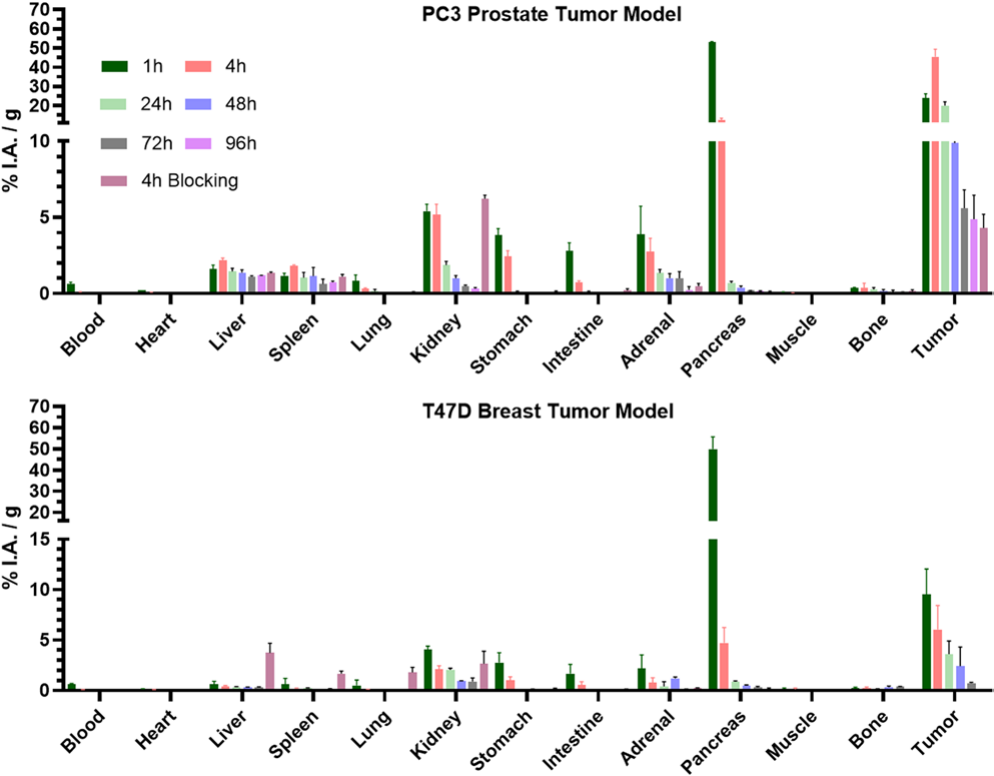
Biodistribution data of [^47^Sc]Sc-LF1 in PC3- and T47D-mice at 1, 4, 24, 48, 72 and 96 h p.i along with blocking studies data at 4 h p.i. Data have been calculated as %I.A./g of tissue and are presented as mean ± SD (*n* = 3–4)

The biodistribution profiles of [^47^Sc]Sc-LF1 in PC3- and T47D-mice are summarized in Fig. 2; Table 1, and Tables S1 and S2. In both models, [^47^Sc]Sc-LF1 showed rapid blood clearance, with blood levels of ~ 0.7%I.A./g at 1 h p.i., resulting in high tumor-to-blood ratios at all time points. The differences in GRPR-expression were reflected on tumor uptake, which was significantly higher in PC3-mice, reaching 24.1 ± 2.1 and 45.4 ± 3.9%I.A./g at 1 and 4 h p.i., respectively, followed by a decline to 4.9 ± 1.6%I.A./g at 96 h. In contrast, T47D-tumors showed lower uptake: 9.5 ± 2.5 and 6.1 ± 2.4%I.A./g at 1 and 4 h, dropping to 0.7 ± 0.1%I.A./g by 72 h. Both models showed high initial uptake in the GRPR-rich pancreas (~ 52%I.A./g at 1 h p.i.), with faster clearance in T47D-mice (10-fold drop by 4 h vs. 4.2-fold in PC3-mice). However, the higher PC-tumors uptakes resulted in increased tumor-to-pancreas ratios in PC3- compared to T47D-mice (> 25 at later time points for PC3 vs. >4 in T47D).

**Table 1:** Tumor-to-tissue ratios of [^47^Sc]Sc-LF1 at 1, 4, 24, 48, 72 and 96 h p.i

	1 h	4 h	24 h	48 h	72 h	96 h
PC3-mice						
Tumor/Blood	44 ± 15	334 ± 89	802 ± 432	˃1000	˃1000	˃1000
Tumor/Pancreas	0.5 ± 0.1	3.3 ± 0.8	30 ± 6	26 ± 7	30 ± 8	30 ± 7
Tumor/Liver	17 ± 3	19 ± 3	14 ± 2	7 ± 1	6 ± 2	3.9 ± 1.3
Tumor/Kidney	5.3 ± 1.5	8 ± 2	11 ± 2	10 ± 2	11 ± 2	14 ± 3
Tumor/Muscle	271 ± 90	˃1000	˃1000	˃1000	˃1000	˃1000
T47D-mice						
Tumor/Blood	16 ± 5	78 ± 25	360 ± 59	411 ± 127	488 ± 271	
Tumor/Pancreas	0.2 ± 0.1	1.5 ± 0.4	4.3 ± 0.6	4.9 ± 2.9	4.0 ± 0.3	
Tumor/Liver	15 ± 1	14 ± 4	11 ± 0.7	7.5 ± 4.9	2.6 ± 0.6	
Tumor/Kidney	2.4 ± 0.8	2.9 ± 0.5	1.7 ± 0.2	2.5 ± 1.3	1.2 ± 0.3	
Tumor/Muscle	52 ± 10	44 ± 15	49 ± 8	53 ± 8.9	39 ± 16	

[^47^Sc]Sc-LF1 was cleared primarily via the urinary route in both models, with low liver uptake (< 2%I.A./g in PC3, < 1%I.A./g in T47D). Kidney uptake was higher in PC3-mice at early time points (5.4 ± 0.4 and 5.2 ± 0.7%I.A./g at 1 and 4 h p.i.) compared to T47D-mice (4.0 ± 0.4 and 2.1 ± 0.3%I.A./g). In both cases, kidney activity declined to < 2%IA/g by 24 h. Consequently, tumor-to-kidney ratios were more favorable in PC3-mice (≥ 10 at 24 h) than in T47D. Differences in non-target tissues such as muscle and gastrointestinal tract were observed, with T47D-mice showing slightly higher non-specific uptake. GRPR specificity was confirmed by the blocking studies, where tumor uptake was reduced by > 90% in GRPR-positive tissues.

### In vivo protein binding/metabolic stability studies in murine plasma

**Fig. 3:**
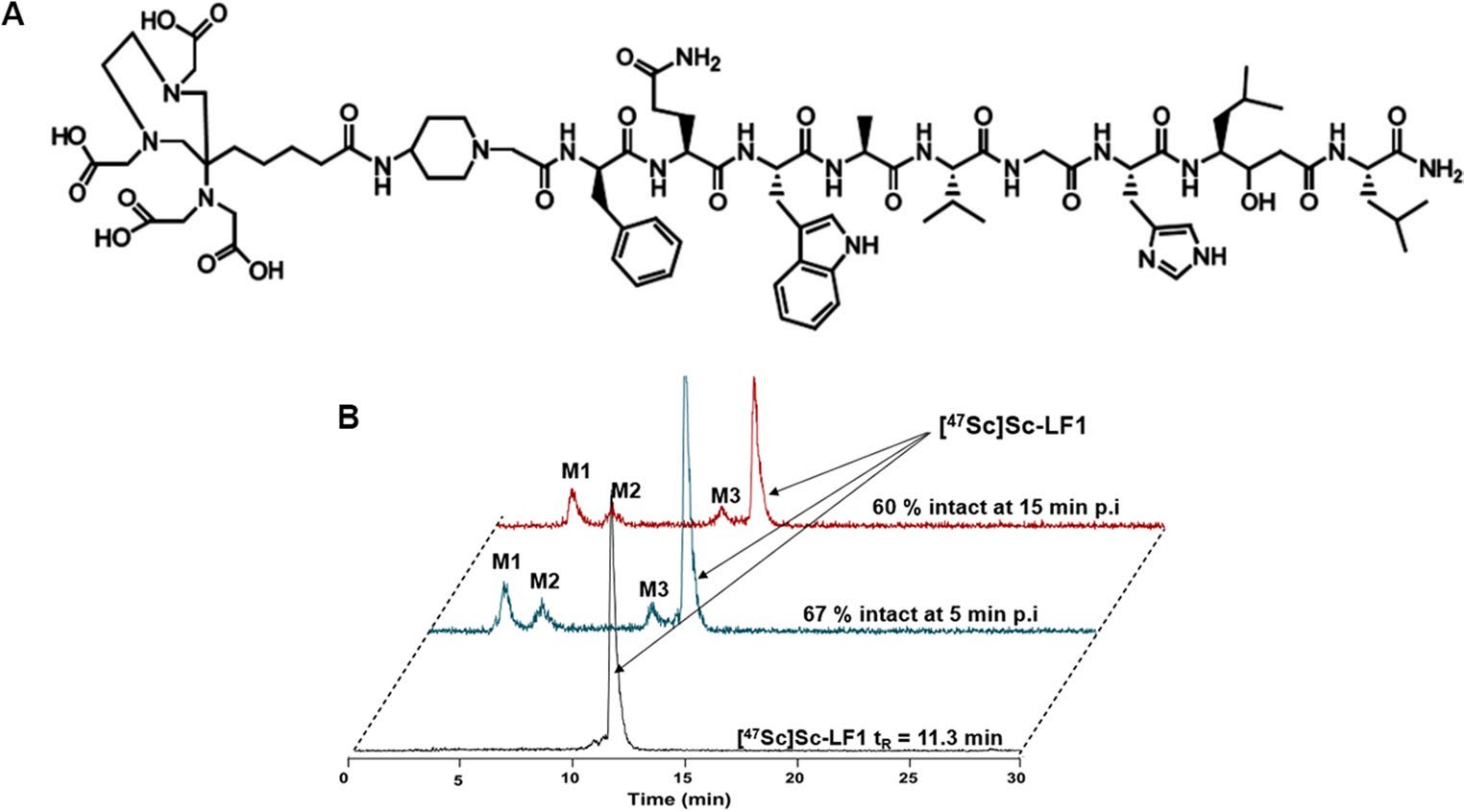
**(A)** Chemical structure of LF1, **(B)** Representative HPLC-chromatograms of blood samples collected at 5 (blue) and 15 min (red) p.i., revealing three metabolites (M1, M2, M3). The reference chromatogram of [^47^Sc]Sc-LF1 before injection in healthy mice is shown in black

Plasma analysis in healthy mice showed low plasma protein binding of [^47^Sc]Sc-LF1 (3.2% at 5 min and 3.7% at 15 min p.i.) Fast proteolytic cleavage was observed, with three metabolites detected (Fig. 3B). The intact [^47^Sc]Sc-LF1 decreased from 67.6 ± 2.2% at 5 min to 60.2 ± 0.9% at 15 min p.i.

### Blood and organs clearance kinetics

**Fig. 4:**
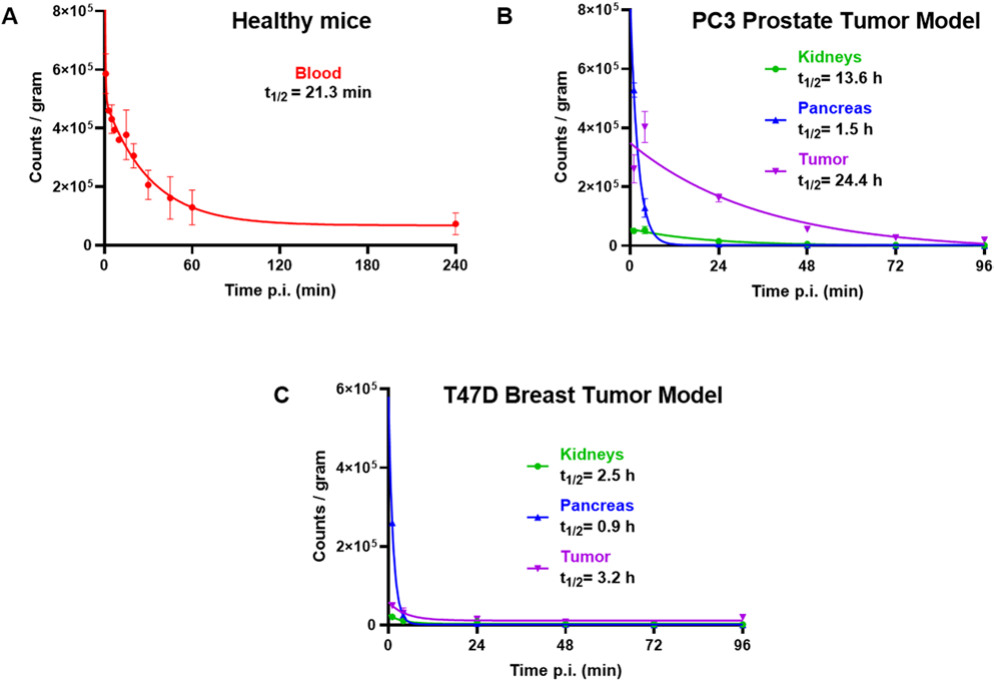
**(A)** Blood clearance of [^47^Sc]Sc-LF1, **(B)** and **(C)** Pharmacokinetic modelling of [^47^Sc]Sc-LF1 clearance from tumor (violet), pancreas (blue) and kidneys (green) based on biodistribution data from PC3- and T47D-mice

The effective half-life of [^47^Sc]Sc-LF1 in blood was 21.3 min, demonstrating its rapid systemic clearance (Fig. 4A). Pancreas exhibited an effective half-life of 1.4 and 0.9 h in PC3- and T47D-mice, respectively (Fig. 4B and C), reflecting moderate retention of the radiotracer. Tumors exhibited the highest effective half-lives of 24.4 h for PC3- and 3.2 h for T47D-mice (Fig. 4B and C). Kidneys showed an effective half-life of 13.6 and 2.5 h for PC3- and T47D-mice, respectively (Fig. 4B and C).

### Area under the time-activity curves (AUC)

**Fig. 5:**
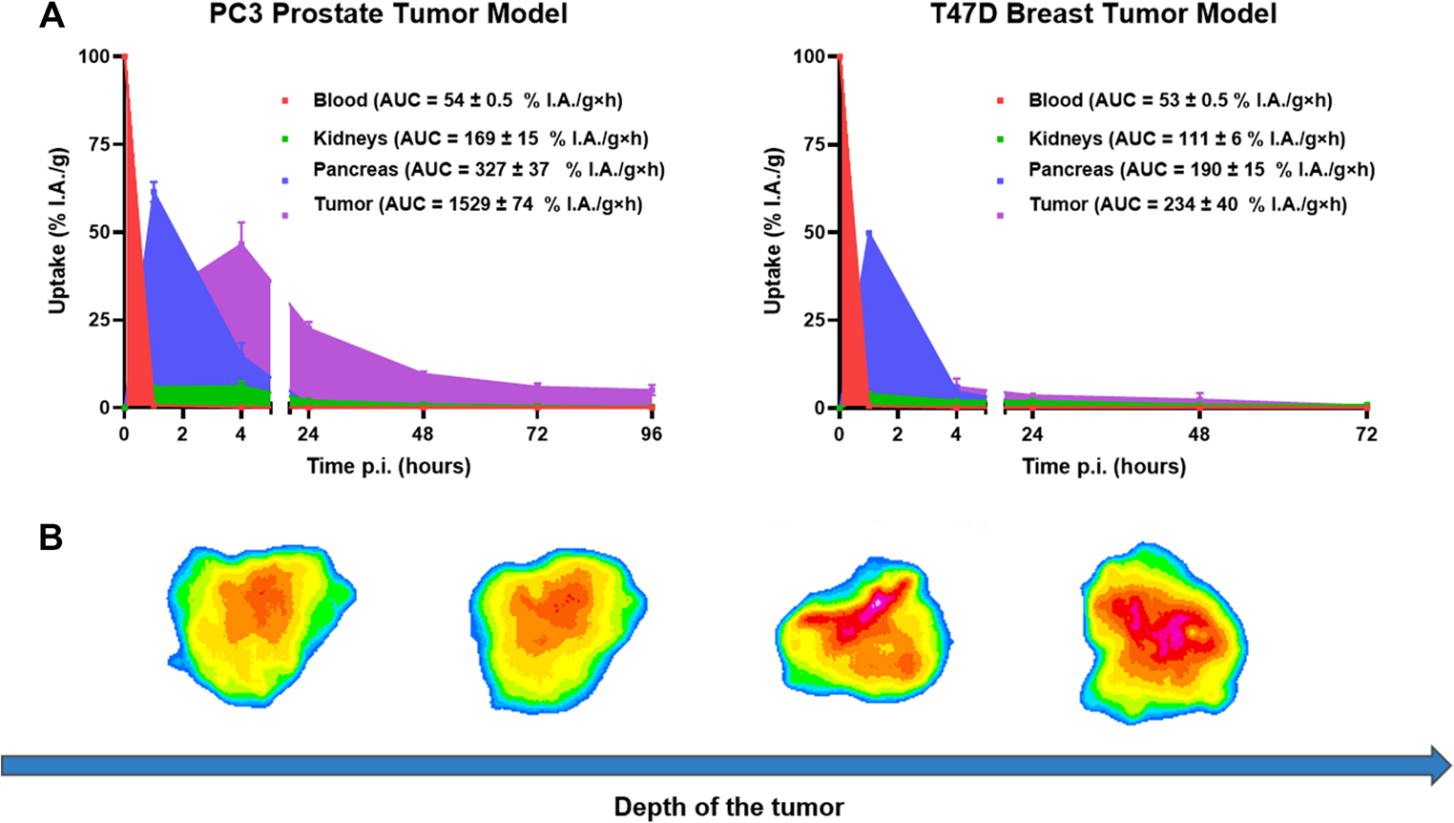
**(A)** AUCs of [^47^Sc]Sc-LF1 in PC3-and T47D-mice for tumor (violet), pancreas (blue), blood (red) and kidneys (green). The curves were generated from the biodistribution data (non-corrected) and given as mean ± standard deviation and in % I.A./g×h. **(B)** Ex vivo autoradiography of PC3 tumor sections from mice injected with [^47^Sc]Sc-LF1 and collected at 1 h p.i

Figure 5A shows the AUCs from non-corrected biodistribution data for tumor, blood, kidneys, and pancreas in both models. In PC3-mice, [^47^Sc]Sc-LF1 showed favorable in vivo behavior with high tumor retention (AUC:1529 ± 74%I.A./g×h), about 5 times higher than in the pancreas (327 ± 37%I.A./g×h) and kidneys (169 ± 15%I.A./g×h). In T47D-mice, tumor AUC was lower (234 ± 40%I.A./g×h) compared to PC3-mice, however, it still exceeded that of the pancreas (190 ± 15%I.A./g×h) and kidneys (111 ± 6%I.A./g×h). Blood AUC dropped rapidly after injection in both models (54 ± 0.5%I.A./g×h).

### Ex vivo autoradiography

The autoradiography analysis of [^47^Sc]Sc-LF1 in PC3-mice revealed a heterogeneous intratumoral distribution of radioactivity (Fig. 5B). The majority of the signal was localized to the central regions of the tumor, with markedly higher accumulation observed in the deeper tumor tissue compared to peripheral areas.

### Small-animal SPECT/CT imaging

**Fig. 6:**
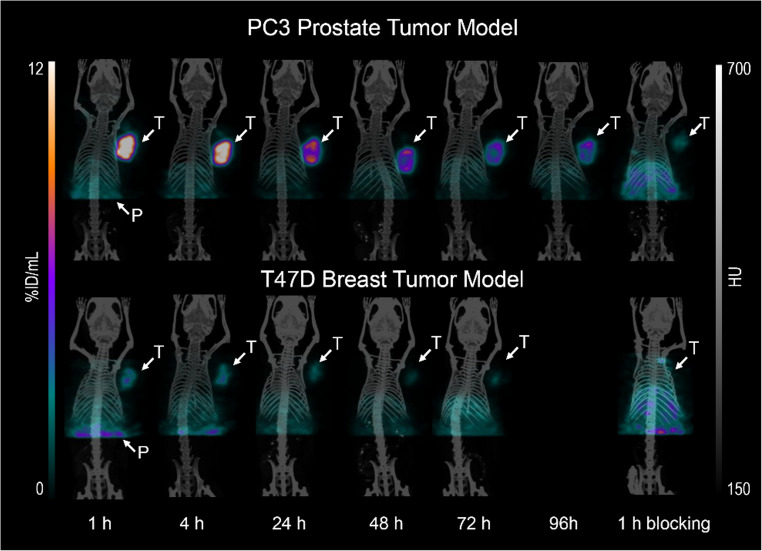
SPECT/CT images of PC3- and T47D-mice upon injection of [^47^Sc]Sc-LF1 at 1, 4, 24, 48, 72 and 96 h along with blocking studies at 4 h after injection. White arrows indicate tumor (T) and pancreas (P) uptake

SPECT/CT images were acquired at 1, 4, 24, 48 and 72 h p.i. of [^47^Sc]Sc-LF1 in T47D- and until 96 h in PC3- mice (Fig. 6). The SPECT images were in agreement with the results from the biodistribution studies. Specific and fast tumor and pancreas uptake was detected at 1 h p.i., and a considerably faster washout of the radioactivity was observed from the pancreas compared to the tumor. Already at 4 h p.i., the initial accumulated activity in the pancreas was washed out, and mainly the tumor was well visible even at 96 h p.i. The specificity of the tumor uptake was verified by blocking experiments.

### In vivo monotherapy with [^47^Sc]Sc-LF1

**Fig. 7:**
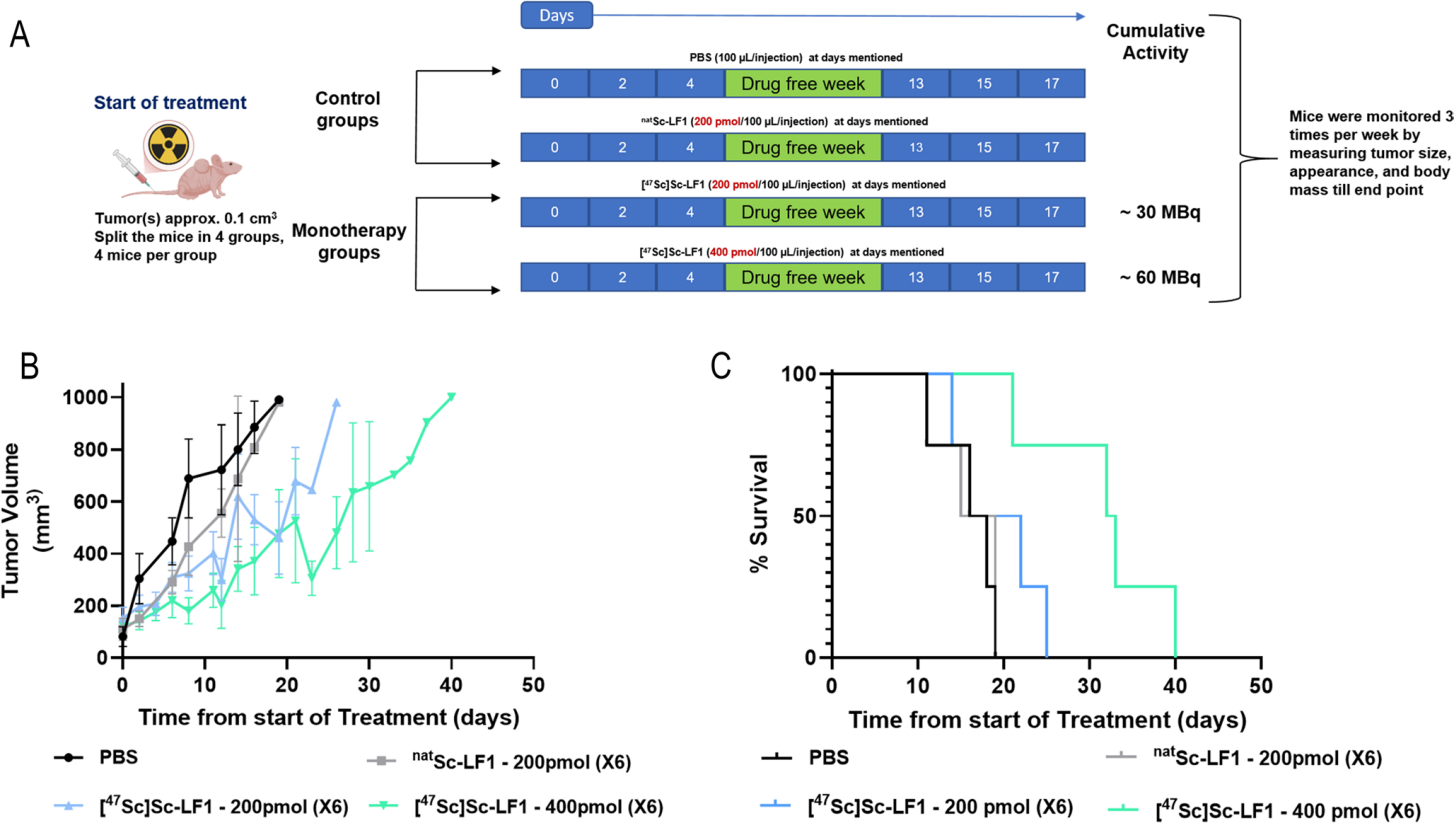
**(A)** Schematic representation of [^47^Sc]Sc-LF1 monotherapy in PC3-mice. **(B)** Tumor volume (mm^3^) changes over the treatment period. **(C)** Survival rates over time in the different monotherapy groups

PC3-tumors in the control groups (PBS or ^nat^Sc-LF1) progressed rapidly, with all mice sacrificed within 20 days (Fig. 7). Mice receiving the lower dose of [^47^Sc]Sc-LF1 (30 MBq over six doses) showed slightly improved tumor control, reaching endpoints within 25 days. The high-dose group (60 MBq over 6 doses) exhibited significant tumor suppression, with volumes staying below 500 mm^3^ for at least 25 days and endpoints reached within 40 days. Tumor growth remained comparatively slow and controlled over an extended period.

The survival analysis reflected these trends: median survival was 17, 17, and 18.5 days for PBS, ^nat^Sc-LF1, and the low-dose groups, respectively (*p* > 0.5) and 32 days for the high-dose group (*p* < 0.01). Body weight remained stable across all groups.

###  In vivo combination therapy with [^47^Sc]Sc-LF1 and everolimus

**Fig. 8:**
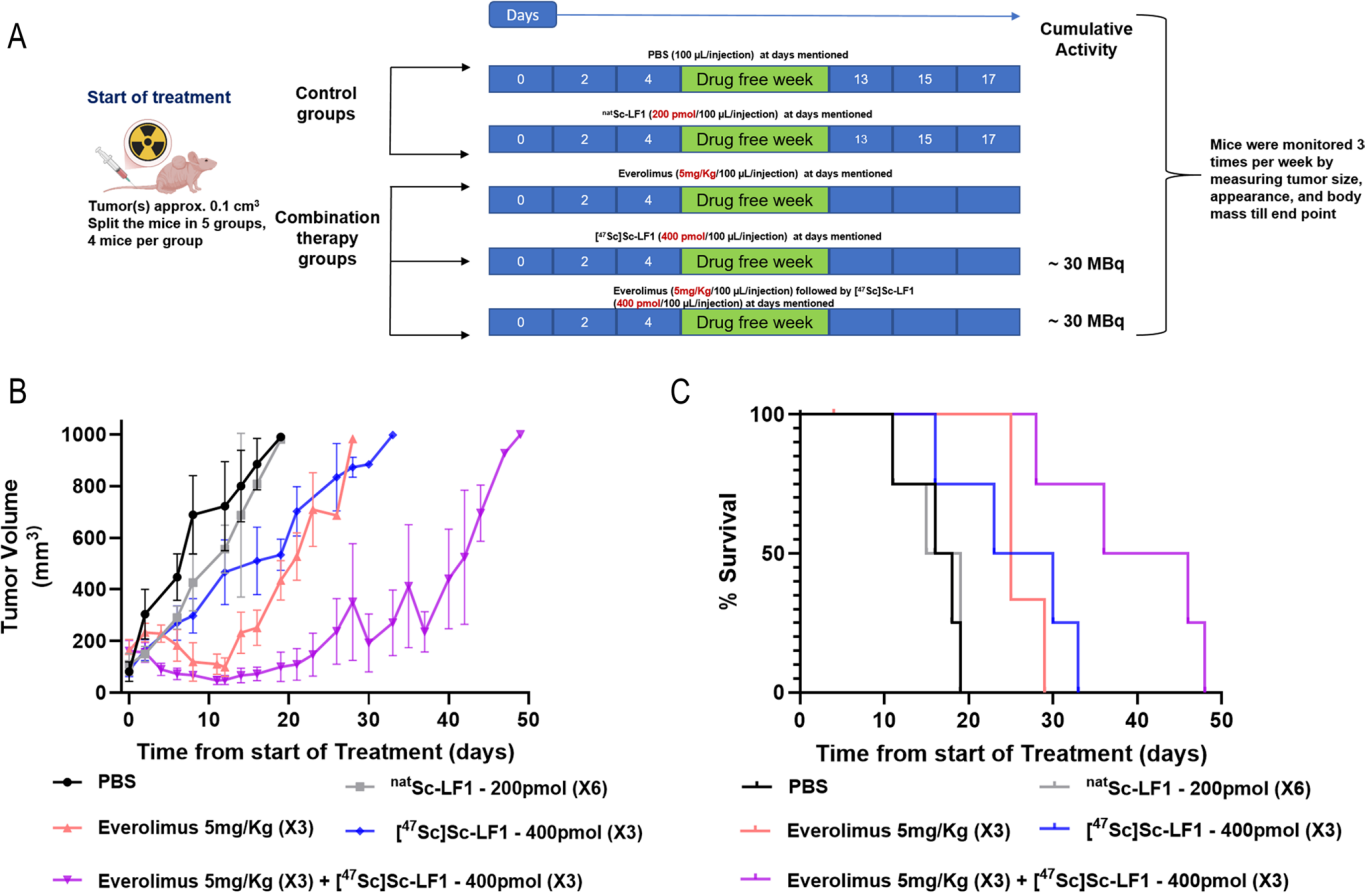
**(A)** Schematic representation of the combination therapy. **(B)** Tumor volume (mm^3^) changes over the treatment period. **(C)** Survival rates over time in the different monotherapy groups

This study evaluated the synergistic effect of everolimus or [^47^Sc]Sc-LF1 alone, and if/how the effect of [^47^Sc]Sc-LF1 on tumor progression was enhanced when combined with everolimus. While PBS and ^nat^Lu-LF1 controls showed rapid tumor growth (> 800 mm^3^ by day 15), everolimus alone moderately delayed tumor growth, keeping tumor volume under 500 mm^3^ for 20 days. The combination therapy (everolimus and [^47^Sc]Sc-LF1, 30 MBq) was most effective, maintaining tumor volumes below 500 mm^3^ for over 40 days, indicating a strong synergistic effect. (Fig. 8). The survival analysis confirmed this benefit, showing a median survival of 41 days for the combination group versus 17 days for the control group (*p* < 0.01). Everolimus alone and the fractionated [^47^Sc]Sc-LF1 (30 MBq) dose extended the survival to ~ 25 and ~ 27days (*p* > 0.5), respectively.

### Morphological assessment

There was no evidence of treatment-related toxicity at any of the doses administered in this study. This was evaluated through multiple parameters, including regular monitoring of animal appearance, body weight measurements taken at defined intervals, and microscopic tissue examination of organs (kidneys and pancreas) harvested at the study endpoint. Mice tolerated three doses of everolimus well, with no observable side effects based on physical condition or weight fluctuations. Similarly, no obvious lesions were detected in any of the collected tissues from mice treated with monotherapy and combination therapy (Table S3). These findings indicate that the dosing regimens used in both the monotherapy and combination therapy groups were safe and well-tolerated.

## Discussion

The [^44^Sc]Sc/[^47^Sc]Sc-GRPR pair presents a promising theranostic approach for prostate and breast cancer, with several advantages over other GRPR-targeting radiopharmaceuticals. Unlike common pairs, like [^68^Ga]Ga/[^177^Lu]Lu, [^44^Sc]Sc/[^47^Sc]Sc, they share identical coordination chemistry, enabling matched pharmacokinetics for more accurate dosimetry and personalized therapy [11, 15, 16]. [^44^Sc]Sc, a positron emitter with a 3.97 h half-life, offers superior PET imaging quality and flexibility for delayed scans.

The selection of AAZTA as a chelator in this study was driven by its highly favorable coordination chemistry and its suitability for theranostic applications. AAZTA allows fast and efficient radiolabeling under mild conditions, typically at room temperature, while forming highly stable complexes with scandium radioisotopes. These characteristics enabled the straightforward and reproducible synthesis of both [^44^Sc]Sc-LF1 and [^47^Sc]Sc-LF1 under identical, mild conditions [20, 21]. The strong kinetic and thermodynamic stability of Sc-AAZTA complexes provides an advantage over other chelators, facilitating reliable in vivo performance and supporting translation to both diagnostic ([^44^Sc]Sc-LF1) and therapeutic ([^47^Sc]Sc-LF1) applications. [^47^Sc]Sc emits moderate-energy β⁻ particles, effectively targeting tumors while sparing nearby healthy tissue. It represents a promising alternative to lutetium, combining favorable physical decay properties with fast complexation kinetics and high complex stability when coordinated to AAZTA [15, 20, 21]. Moreover, the matched ^44^Sc/^47^Sc pair offers distinct advantages including deeper tissue penetration, superior image quality and quantitative accuracy in PET imaging, lower background signal in SPECT, and improved dosimetry precision (Table S5) [15]. Consistent with these observations, our previous studies using [^177^Lu]Lu-LF1 [22] also demonstrated high in vitro and in vivo stability and very low protein binding, confirming AAZTA’s suitability for both lutetium and scandium coordination. While we and others have acknowledged that AAZTA is not the optimal chelator for gallium-68 [17, 20, 21], it performs exceptionally well with trivalent metals such as Sc and Lu. Direct comparison of LF1 (AAZTA-chelated) with RM2 (DOTA-chelated), which share the same peptide motif but differ only in the chelator, revealed comparable in vivo stability; however, LF1 exhibited significantly higher tumor uptake and improved tumor-to-background ratios [8, 17, 20, 22].

The distant location of the ^44^Sc/^44^Ti generator from our facility resulted in low scandium-44 activity at the start of labeling. Therefore, we only conducted a few proof-of-concept radiolabelings with [^44^Sc]Sc^3+^, and proceeded with [^47^Sc]Sc^3+^, given their equivalent coordination chemistry.

We evaluated the targeting potential of [^47^Sc]Sc-LF1 using two tumor models with different GRPR-expression: PC3 (high GRPR-expression) and T47D (moderate GRPR-expression). PC3 prostate cancer cells enabled assessment of maximal tracer binding and treatment efficacy, while T47D breast cancer cells tested the tracer’s ability to distinguish tumors based on receptor levels. As expected, [^47^Sc]Sc-LF1 showed strong GRPR selectivity and high affinity for both cell lines. PC3 cells had higher B_max_ values and, in vivo, exhibited threefold greater tumor uptake than T47D, confirmed by SPECT/CT and biodistribution studies (Figs. 2 and 6).

[^47^Sc]Sc-LF1 showed low internalization in PC3 and T47D cells, consistent with the behavior of GRPR antagonists, which typically remain on the cell surface due to limited receptor-mediated endocytosis [8, 17, 23–25]. Previous studies found that RM2 (10 µM) does not induce calcium mobilization or receptor internalization in PC3 cells, confirming its antagonist profile [17, 23]. Our results indicate that AAZTA^5m^ functionalization did not affect the statine-based motif, and LF1 retains its GRPR-antagonistic properties.

In comparison with previously reported GRPR-targeting radioligands, [^47^Sc]Sc-LF1 demonstrated favorable pharmacokinetic and biodistribution characteristics (Table S4) [8–10, 22–29]. In PC3 tumor bearing mice, [^47^Sc]Sc-LF1 exhibited high and persistent tumor uptake (24.1 and 19.9%IA/g at 1 and 24 h, respectively; T_1/2_=22.4 h), which was only moderately lower than that of [^177^Lu]Lu-LF1 (42 and 18%IA/g) and clearly higher than values reported for [^177^Lu]Lu-NeoB (9 and 8%IA/g), [^177^Lu]Lu-AMTG (14 and 11%IA/g), and [^177^Lu]Lu-RM2 (12 and 8%IA/g). [^47^Sc]Sc-LF1 showed rapid blood clearance with an effective half-life of 21.3 min, thereby minimizing systemic exposure and reducing the risk of off-target radiation. Despite its initially high pancreatic uptake (52.9%IA/g at 1 h), activity decreased sharply to 0.7%IA/g at 24 h, indicating efficient clearance from non-target GRPR-positive tissues and improved tumor-to-background ratios over time. Renal uptake (5.4 and 1.9%IA/g) was moderate and comparable to that of [^177^Lu]Lu-AMTG and [^177^Lu]Lu-RM2, while hepatic accumulation remained low (1.6 and 1.5%IA/g), similar to [^177^Lu]Lu-LF1 [8, 11, 22, 23]. In the T47D breast cancer model, [^47^Sc]Sc-LF1 exhibited tumor uptake and pharmacokinetics comparable to those of [^111^In]In-JMV4168, with approximately 40–45% of the tumor-associated activity clearing within 24 h, indicating favorable retention for therapeutic applications [28]. Overall, [^47^Sc]Sc-LF1 combines high and sustained tumor accumulation with rapid systemic and non-target clearance, resulting in a biodistribution profile superior to or comparable with other GRPR-targeting radioligands. These features, together with the physical decay characteristics of ^47^Sc, support its potential as a promising theranostic agent within the matched ^44^Sc/^47^Sc radionuclide pair.

[^47^Sc]Sc-LF1 showed excellent radiochemical stability, however, it underwent rapid enzymatic degradation in vivo due to neprilysin (NEP), a zinc-dependent metallopeptidase known to limit the therapeutic efficacy of the statine-based GRPR-radioantagonists [29–31]. This cleavage produced three main metabolites that increased over time. [^47^Sc]Sc-LF1`s fast blood clearance suggests that the generated metabolites are small, hydrophilic fragments that are quickly eliminated, without contributing to background uptake. Similar metabolic behavior has been observed for [^68^Ga]Ga-RM2 and [^177^Lu]Lu-LF1 [22, 29].

The high and sustained tumor uptake of [^47^Sc]Sc-LF1, along with its rapid clearance from blood and non-target organs, observed in both biodistribution and imaging studies, supports its potential therapeutic value. A fractionated dosing regimen was used in PC3-mice, an established approach in radionuclide therapy that optimizes tumor targeting while minimizing damage to healthy tissue. The injected mass per session was based on prior studies showing it provides effective GRPR targeting without receptor saturation and aligns with safe, effective levels reported for [^177^Lu]Lu-RM2 and [^177^Lu]Lu-LF1 [22, 32]. In monotherapy, [^47^Sc]Sc-LF1 showed a clear dose-dependent therapeutic effect, with higher doses achieving better tumor control and prolonged survival (Fig. 7). In line with our previous findings with [^177^Lu]Lu-LF1 [22], the present study with [^47^Sc]Sc-LF1, similarly demonstrated a clear dose-dependent therapeutic effect in monotherapy settings. In both cases, administration of higher or fractionated doses resulted in improved tumor control and significantly prolonged survival in PC3-mice. Although the absolute administered activities differed due to distinct initial activities of [^177^Lu]LuCl_3_ and [^47^Sc]ScCl_3_ (in case of [^177^Lu]Lu-LF1 the injected activities were higher compared to [^47^Sc]Sc-LF1), the overall therapeutic trends remained consistent, highlighting the robustness and reproducibility of LF1 as a versatile GRPR-targeted theranostic platform.

The combination of mTOR inhibition with TRT offers a promising strategy to enhance therapeutic efficacy in cancer treatment. The mTOR pathway is frequently dysregulated in tumors, promoting survival, proliferation, and resistance to therapy, including enhanced DNA repair following radiation [33]. Inhibiting this pathway can sensitize tumor cells to TRT by disrupting these protective mechanisms. Everolimus, a clinically approved mTOR inhibitor with well-characterized pharmacological properties, was chosen for our study due to its established safety profile and effectiveness in targeting mTOR signalling [34]. Our results demonstrated that combining everolimus with [^47^Sc]Sc-LF1 significantly improved tumor control and prolonged survival in treated mice (Fig. 8). Importantly, the combination of fractionated TRT with the mTOR inhibitor everolimus further improved the therapeutic outcome. In both radionuclide systems, the combination strategy enhanced tumor growth control and prolonged survival compared to monotherapy alone. Both lutetium-177 and scandium-47 demonstrated clear therapeutic efficacy. Particularly, the modified [^47^Sc]Sc-LF1 combination protocol conferred a modest but measurable survival benefit compared to [^177^Lu]Lu-LF1. In the [^47^Sc]Sc-LF1 regimen, everolimus was administered concomitantly with the radioligand, whereas in the [^177^Lu]Lu-LF1 protocol, everolimus was given for three consecutive days prior to radioligand administration [22]. This observation highlights the potential of [^47^Sc]Sc as not only a diagnostic partner to [^177^Lu]Lu but also as a therapeutic radionuclide with promising efficacy. These findings suggest that fractionated radionuclide therapy in combination with molecularly targeted agents such as everolimus represents a rational and innovative approach to improve treatment outcomes in prostate cancer. The synergistic interaction between LF1-based TRT and mTOR inhibition provides a strong rationale for further preclinical and translational evaluation.

In conclusion, the true theranostic pair [^44^Sc]Sc/[^47^Sc]Sc-LF1, shows strong potential in the management of GRPR-expressing tumors, and the results obtained so far support its further development for targeted radionuclide therapy, including dosimetry studies and future clinical evaluation.

## Supplementary Information

Below is the link to the electronic supplementary material.

Supplementary Material 1 (DOCX 532 KB)

## Data Availability

The datasets generated during and/or analysed during the current study are available from the corresponding author on reasonable request.
