# Supplementary material for: A true theranostic pair – 44/47Sc-labeled GRPR antagonist shows great promise for managing prostate and breast cancer
Source: Eur J Nucl Med Mol Imaging. 2025 Nov 11;53(4):2554–65. doi: 10.1007/s00259-025-07651-y (PMC12920379; doi:10.1007/s00259-025-07651-y)
Supplement: Supplementary file 1 — Supplementary Material 1 (DOCX 532 KB) [file 259_2025_7651_MOESM1_ESM.docx]

**SUPPLEMENTARY MATERIAL**

**A True Theranostic Pair – ^44/47^Sc-Labeled GRPR Antagonist Shows Great Promise for Managing Prostate and Breast Cancer**

Naveen Kumar^1^, Adrianna Bilinska^1,2^, Elena Menéndez^1^, Tilman Läppchen^1^, Euy Sung Moon^3^, Malgorzata Zoltowska^4^, Dariusz Pawlak^4^, Izabela Cieszykowska^4^, Renata Mikolajczak^4^, Frank Rösch^3^, Axel Rominger^1^, Eleni Gourni^1*^

^1^Department of Nuclear Medicine, Inselspital, Bern University Hospital, University of Bern, Bern, Switzerland

^2^Graduate School of Cellular and Biomedical Sciences, University of Bern, Bern, Switzerland

^3^Department of Chemistry—TRIGA site, Johannes Gutenberg University of Mainz, Germany

^4^Radioisotope Centre POLATOM, National Centre for Nuclear Research, Otwock, Poland

***Corresponding author:**

Eleni Gourni

Department of Nuclear Medicine

Bern University Hospital, Switzerland

Rosenbühlgasse 25, 3010 Bern, Switzerland.

Email: eleni.gourni@insel.ch

Tel.: +41 (0)31 664 0507.

**METHODS**

**Reagents and Instrumentation**

All reagents were of the best grade available and were purchased from common suppliers. All culture reagents were from Gibco BRL, Life Technologies (Grand Island, NY). LF1 was synthesized manually using standard Fmoc chemistry as described earlier at Hofstetter et al, EJNMMI Radiopharmacy and Chemistry 2020). The breast carcinoma cancer cell line (T47D, DSMZ-ACC 739, Lot number 4) was acquired from Leibniz Institute DSMZ (DSMZ GmbH, Braunschweig, Germany). The human prostate adenocarcinoma cell line PC3 (CLS-300312, Lot number 816SF) was obtained from Cell Lines Service GmbH (CLS, Eppelheim, Germany) and breast cancer cells T47D were obtained from Leibniz Institute DSMZ GmbH (Braunschweig, Germany). The human serum was commercially available from Sigma (H4522). [^47^Sc]Sc^3+^ was obtained from Radioisotope Centre POLATOM, National Centre for Nuclear Research, Poland. The quality control of the precursor and the radiotracers was performed by analytical reverse-phase high-performance liquid chromatography (RP-HPLC) on an analytical Nucleosil 120-5 column C18 (250 × 4.5 mm) applying a linear gradient of 15–90% solvent B in 30 min at a flow rate of 1 mL/min. (solvent A, 0.1% trifluoroacetic acid (TFA)/water (H2O); solvent B, 0.1%TFA/acetonitrile (ACN). Ultraviolet detection was performed using an Agilent detector at 214 nm. For radioactivity measurement, a Na(TI) well-type scintillation Gina star was used. The radiotracer solutions for the experiments were prepared by dilution with 0.9% NaCl (Bichsel AG, Interlaken, Switzerland). Quantitative γ-counting was performed with a COBRA II γ-system well counter from Packard Instruments (USA). For the SPECT/CT studies, a dedicated SPECT/CT scanner (Albira Si; Bruker Biospin, Ettlingen, Germany) was used. All experiments were carried out twice in triplicate. Mice were purchased from Janvier Labs (Rue du Genest, 53940 Le Genest-Saint-Isle, France), pentobarbital natrium (150mg/kg) from Streuli Pharma SA (Uznach, Switzerland).

**Radiolabeling / Quality Control**

The radiolabeling of [^44^Sc]Sc-LF1 was performed by adding 30 μg of LF1 (approximately 18 nmol) in 750 μL ammonium acetate buffer (0.25 M, pH 4) with activity of [^44^Sc]Sc^3+^ (9 MBq), depending on the availability. The radiolabeling was completed within 20-30 min at 90^o^C. For [^47^Sc]Sc-LF1, radiolabeling was performed by dissolving 5–8 μg of LF1 (approximately 3–5 nmol) in 250 μL HEPES buffer (1.0 M, pH 5.4) and 20 µL of EtOH followed by the anticipated activity of [^47^Sc]Sc^3+^ (20–190 MBq) depending on the experiment. The radiolabeling was completed within 30 min at room temperature. The quality control of [^47^Sc]Sc-LF1 was performed by radio-HPLC and radio thin layer chromatography (radio-TLC) as described in the reagents and instrumentation (Fig. S1). To the radiolabelings which were performed with high amounts of radioactivity, immediately after the completion of the radiolabeling, ascorbic acid was added to the [^47^Sc]Sc-LF1radiolabeling mixture to a final concentration of 20 µg/µL, to prevent autoradiolysis. The radiochemical stability of [^47^Sc]Sc-LF1 was evaluated for a period of 192 h by radio-TLC analysis, as shown in Fig. S2.

**Lipophilicity Studies and protein binding**

The lipophilicity (Log_DOctanol/PBS_, pH 7.4) was estimated by the “shake–flask” method: the labelled conjugate (~20 pmol; 0.1 MBq) was added to a 1:1 mixture of 1-octanol (500 μL) and PBS (500 μL, pH 7.4). The mixture was intermittently vortexed for 1 h to reach the equilibrium and then centrifuged (3000 rpm) for 10 min. From each phase, an aliquot (50 μL) was pipetted out and measured in a gamma counter. Each measurement was repeated five times. Care was taken to avoid cross-contamination between the phases. The partition coefficient was calculated as the average log ratio of the radioactivity in the organic fraction and the PBS fraction.

[^47^Sc]Sc-LF1 (~20 pmol; 0.1 MBq) was incubated with commercially available human serum (0.5 mL) at 37 °C for 60 minutes. When the incubating period was completed, proteins were precipitated with a solution of 1 mL MeOH/ACN (1:1). Centrifugation (10 minutes, 3000 rpm) was performed for the separation of proteins. After careful separation of the two phases, the respective activities were measured in a gamma-counter, followed by determination of the percentage of the radiotracer which binds to the serum proteins.

**Cell Lines**

The human prostate adenocarcinoma cell line PC3 was cultured in Dulbecco's Modified Eagle Medium (DMEM) with low glucose (1 g/L) combined with F-12 Nutrient Mix with GlutaMAX™-I (1:1 mixture ratio). The breast carcinoma cancer cell line T47D was cultured in Dulbecco's Modified Eagle Medium (DMEM) with high glucose (4.5 g/L) supplemented with L-alanyl-L-glutamine dipeptide and sodium bicarbonate. The medium was supplemented with 5-10% fetal bovine serum (FBS), penicillin (100 U/mL) and streptomycin (100 µg/mL). All of the cell lines were cultured at 37 °C and 5% CO_2_.

**Saturation Binding Studies:**

For the receptor saturation analysis, PC3 and T47D cells overexpressing GRP receptor were seeded at a density of 0.8-1 million cells per well in 6-well plates. The cells were incubated overnight with medium (DMEM containing 1% FBS, 100 U/mL penicillin and 100 μg/mL streptomycin). The next day, the medium was removed and the cells were incubated for 30 min at 37 °C with 0.8 mL of fresh medium. Afterwards, the plates were placed on ice for 30 min, followed by incubation with increasing concentrations of ^nat/47^Sc-LF1 (1-100 nM final concentration in the wells; 100 µL of radioligand solution and 100 µL of PBS pH 7.4 were added to the cells with 0.8 mL medium in the well). After the addition of the radioligand, the cells were incubated for 120 min at 4°C. Non-specific binding was determined in the presence of blocking agent H-D-Phe-Gln-Trp-Ala-Val-Gly-His-Sta-Leu-NH_2_ at a final concentration of 10 μM. After the completion of the incubation, the cells were washed twice with ice-cold PBS, followed by solubilization with 1 N NaOH. The cell-associated radioactivity was measured using a gamma counter. Specific binding was plotted against the total molar concentration of the added radiotracer. The K_d_ values and the concentration of the radiotracer required to saturate the receptors (Bmax) were determined by nonlinear regression using GraphPad (Prism 10.2.0 Graph Pad Software). For all of the cell studies, the values are normalized for 1x10^6^ cells per well, and all data are from two independent experiments with triplicates in each experiment.

**Internalization Studies**

For the internalization experiments, PC3 and T47D cells were seeded into 6-well plates. On the day of the experiment, approximately 2.5 pmol (100 µL) of the radiotracer was added to the medium (total volume 1.5 mL) and the cells were incubated (in triplicate) for 0.5, 1, 2, 4 and 6 h at 37 °C, 5% CO_2_. To identify the nonspecific membrane binding and internalization, an excess of blocking agent H-D-Phe-Gln-Trp-Ala-Val-Gly-His-Sta-Leu-NH_2_ (final concentration 10 μΜ) was added to the selected wells. At each time point, the internalization was stopped by putting the plates on ice, removing the medium, and washing the cells twice with ice-cold PBS. To remove the receptor-bound radioligand, an acid wash was carried out twice with a 0.1 M glycine buffer, pH 2.8, for 5 min on ice. Finally, the cells were solubilized with 1 N NaOH. The radioactivity of the culture medium, the receptor-bound, and the internalized fractions were measured in a γ-counter.

**Biodistribution, imaging and therapy studies**

For the biodistribution, imaging and therapy studies, mice were randomly designated to groups based on their tumor sizes to ensure that each group had a similar size distribution. All animal experiments were approved by the local authorities and performed in compliance with the institutional guidelines (animal license number BE63/2021; 33892).

**SPECT/CT studies**

SPECT was acquired using a 159 keV ± 20% energy window and a Cerrobase (Bi-Pb alloy) 3 mm thick collimator, with a pinhole 2 mm thick tungsten lens, 60 projections and 120–720 s per projection. Raw SPECT data were reconstructed using the Ordered Subset Expectation Maximization (OSEM) algorithm for 3 iterations of 5 subsets and a voxel size of 0.25mm. The reconstructed data were corrected for Scandium-47 decay, normalized, and filtered using a Gaussian 3D algorithm with a 2 mm isotropic kernel and generated using PMOD software. The CT was carried out using step-and-shoot mode, employing 45 kVp and 400 μA as settings. The images were reconstructed using the Filtered Back Projection (FBP) algorithm and a voxel size of 0.25 mm. The color scale of the SPECT images was set as 0 to 13% IA/mL, allowing for qualitative comparisons among the images.

**In Vivo Protein Binding / Metabolic Stability Studies in Murine Plasma**

Healthy mice (n = 2) were injected with 200 pmol of [^47^Sc]Sc-LF1 in saline (~6 MBq/0.1 mL) in NaCl 0.9% and sacrificed 5 and 15 min post-injection (p.i.). Blood was immediately transferred to pre-chilled heparinized tubes and centrifuged (5 min, 1700 g, 4 °C) for plasma isolation. The isolated plasma was then transferred into a 2 mL Eppendorf and a double volume of a 1:1 (v/v) ACN:MeOH solution was added to induce the precipitation of the plasma proteins. The proteins were separated by centrifugation (10 min, 9660 g, 4 °C). After careful separation of the two phases (precipitated proteins and supernatant), the respective activities were measured in a γ-counter to determine the percentage of the radiotracer bound to the plasma proteins.

To evaluate the in vivo metabolic stability of [^47^Sc]Sc-LF1 in blood circulation and determine if and to which extent the remaining circulating activity in blood is subjected to enzymatic degradation, samples from the supernatant were analyzed by radio-HPLC (from both tested time points 5 and 15 min p.i.).

**In-vivo monotherapy of PC3 tumor bearing mice using [^47^Sc]Sc-LF1**

Tumor regression studies were carried out in order to evaluate the therapeutic efficacy of [^47^Sc]Sc-LF1. A fractionated dosing scheme was applied in male athymic nude mice subcutaneously implanted with PC3 tumors. When the average tumor size at the start of treatment reached approximately 162 ± 70 mm^3^, the mice were divided into 5 groups with 5 mice in each group. A fractionated therapeutic regimen was implemented. Two groups received 3 injections of [^47^Sc]Sc-LF1 per week on days 0, 2, and 4 of therapy. After a one-week drug-free interval, the same procedure was repeated on days 13, 15, and 17. The total administered activity for these groups was ~30 MBq (in total 1200 pmol) and ~60 MBq (in total 2400 pmol), delivered in doses of 4–5 MBq and 10–11 MBq (corresponding to 200 and 400 pmol per dose, respectively). Two control groups were included in the study: one received ^nat^Sc-LF1(in total 1200 pmol) in 100 µL of NaCl 0.9%, and the other received PBS (100 µL). Both control groups were administered their respective treatments on the same days as the [^47^Sc]Sc-LF1 treated groups. The mice were monitored 3 times per week by measuring tumor size and their body weight. As ending point was set when tumors reach a volume of 1.0 cm^3^ or 150 days if the therapy is successful and the above tumor size has not been reached. Tumor size was measured with callipers in 2 dimensions, and tumor volume was calculated assuming an elliptical shape. Tumor volume at each time point was calculated as: Width × (Length)^2^ × 0.5. Body weight of the animals was measured 3 times per week to observe the acute toxicity associated with the treated groups compared to the control animals. Mice were euthanized if they experienced a weight loss exceeding 15% of their initial body weight or if tumor volume surpassed 1.0 cm³ prior to the study endpoint.

**Morphological assessment**

Frozen tissue samples (kidney, pancreas and tumor) were sectioned at 7 µm thickness using a cryostat maintained at −20°C to −25°C and mounted onto microscope glass slides. Sections were air-dried at room temperature for 15 minutes and subsequently fixed in 4% paraformaldehyde for 10 minutes. After rinsing in phosphate-buffered saline, slides were stained with hematoxylin for 5 minutes, rinsed in running tap water and differentiated in 1% acid alcohol followed by bluing in ammonia water. Sections were then counterstained with eosin for 1 minute and briefly washed in 70% ethanol to remove excess stain. Slides were dehydrated through graded ethanol (70%, 95%, 100%) and mounted with a permanent mounting medium. Stained sections were examined using a light microscope.

**RESULTS**

**Quality Control and Radiochemical Stability**

Radio-TLC analysis was performed with aluminium-coated silica gel 60 F254 plates from Merck as the stationary phase and citrate buffer 0.1 M at pH 5 as a mobile phase. In this chromatographic system, the radiolabeled peptide remains immobilized at the starting point, while free lutetium-44/47 moves with the mobile phase.

RP-HPLC analysis was performed using a linear gradient of eluent A (0.1 % (v/v) TFA in water) and eluent B (0.1 % (v/v) TFA in acetonitrile) applied according to the following method: 0-3 min (15 % B), 3-21 min (15-90 % B), 21-25 min (90 % B), 25-25.1 min (90-15 % B) and 25.1-30 (15% B).


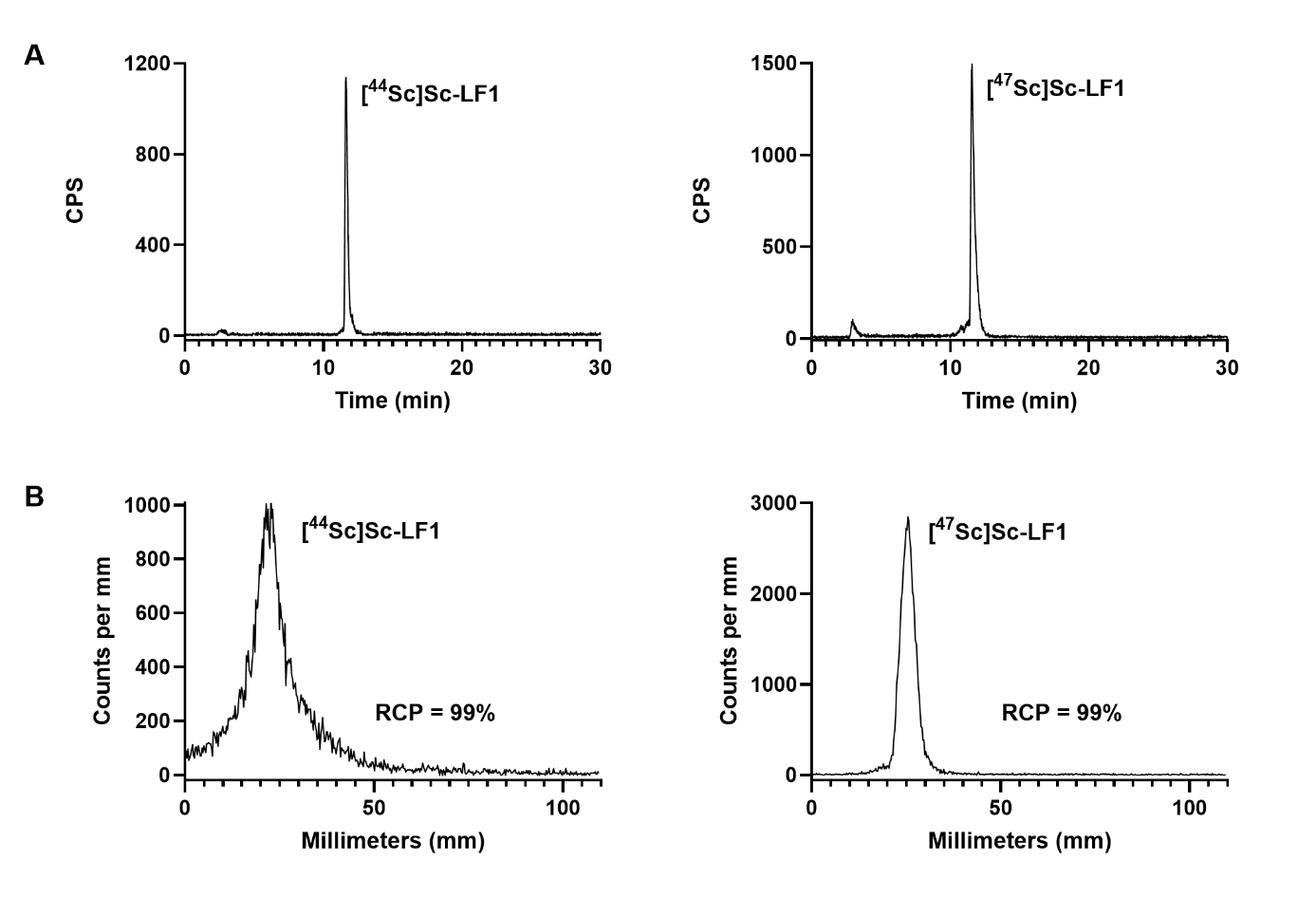


**Figure S1.** **A.** Radio-HPLC profile of [^44^Sc]Sc-LF1 and [^47^Sc]Sc-LF1 performed using a linear gradients of eluent A (0.1 % (v/v) TFA in water) and eluent B (0.1 % (v/v) TFA in acetonitrile) applied according to the following method: 0-3 min (15 % B), 3-21 min (15-90 % B), 21-25 min (90 % B), 25-25.1 min (90-15 % B) and 25.1-30 (15% B). **B.** Radio-TLC profile of [^44^Sc]Sc-LF1 and [^47^Sc]Sc-LF1 performed 5 min post-labeling with aluminium-coated silica gel as the stationary phase and sodium citrate buffer (0.1 M, pH 5.4) as mobile phase.


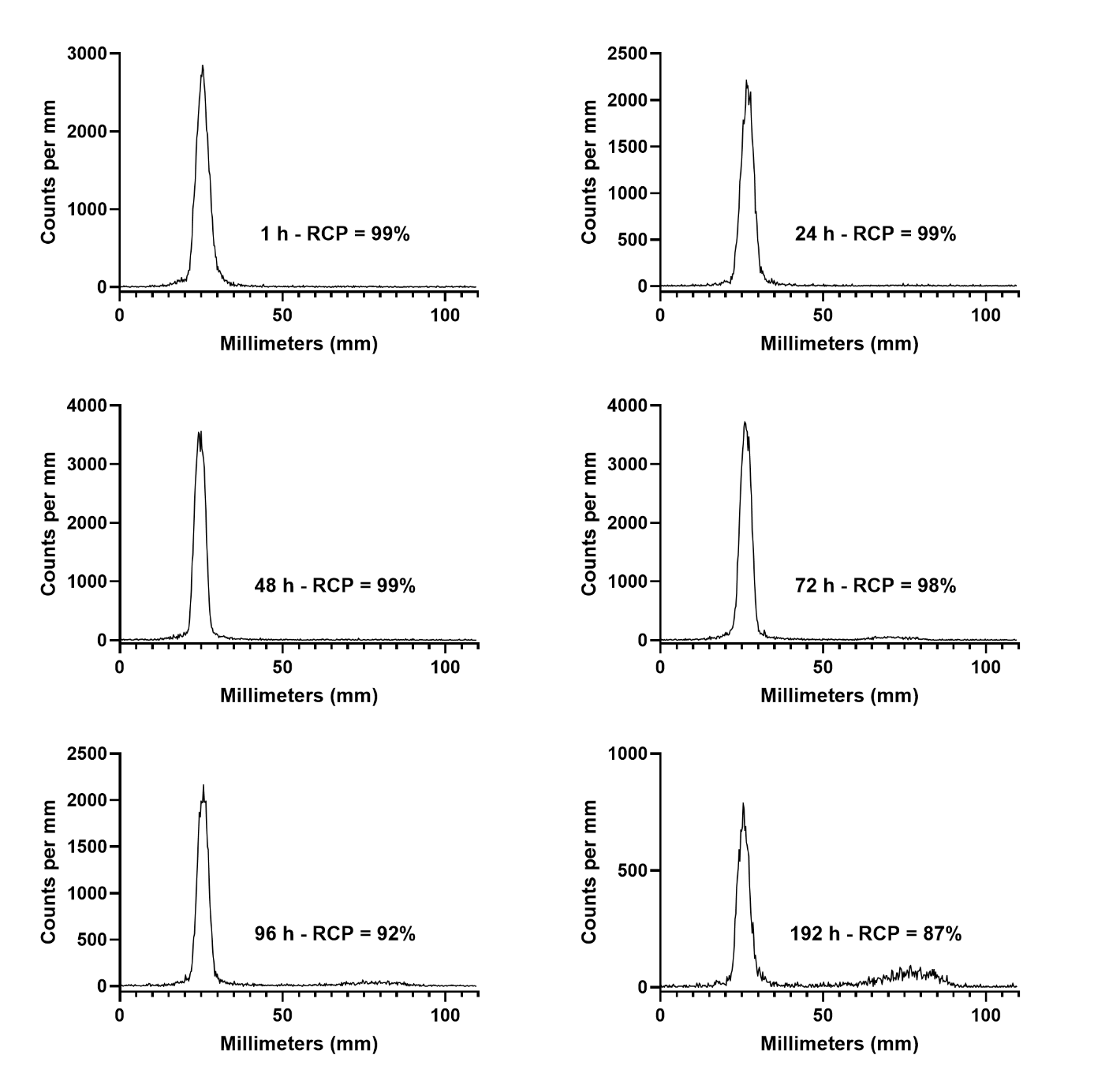


**Figure S2.** Radio-TLC profiles of [^47^Sc]Sc-LF1 (R_f_ = 0) indicating the radiochemical purity (RCP) at several time points after radiolabeling (0, 7, 24, 48, 72 h and 8 d) expressed as percentage of intact tracer detected over total radioactivity.

**Biodistribution Data**

**Table S1:** Biodistribution data of [^47^Sc]Sc-LF1 in PC3 xenografts.

| **Organ** | **1 h** | **4 h** | **24 h** | **48 h** | **72 h** | **96 h** | **4 h blocked** |
| --- | --- | --- | --- | --- | --- | --- | --- |
| Blood | 0.7±0.1 | 0.1±0.0 | 0.0±0.0 | 0.0±0.0 | 0.0±0.0 | 0.0±0.0 | 0.0±0.0 |
| Heart | 0.2±0.0 | 0.1±0.0 | 0.1±0.0 | 0.0±0.0 | 0.0±0.0 | 0.0±0.0 | 0.1±0.0 |
| Liver | 1.6±0.2 | 2.0±0.2 | 1.5±0.2 | 1.4±0.2 | 1.1±0.1 | 1.2±0.0 | 1.3±0.1 |
| Spleen | 1.2±0.2 | 1.8±0.0 | 1.0±0.4 | 1.1±0.6 | 0.6±0.3 | 0.7±0.1 | 1.1±0.1 |
| Lung | 0.8±0.4 | 0.3±0.1 | 0.2±0.1 | 0.1±0.1 | 0.1±0.0 | 0.0±0.0 | 0.1±0.0 |
| Kidneys | 5.4±0.4 | 5.2±0.7 | 1.9±0.2 | 1.0±0.2 | 0.5±0.1 | 0.3±0.1 | 6.2±0.2 |
| Stomach | 3.8±0.4 | 2.4±0.4 | 0.1±0.0 | 0.1±0.0 | 0.0±0.0 | 0.0±0.0 | 0.1±0.0 |
| Intestines | 2.8±0.5 | 0.7±0.1 | 0.1±0.0 | 0.1±0.0 | 0.0±0.0 | 0.0±0.0 | 0.2±0.1 |
| Adrenal | 3.9±1.9 | 2.8±0.9 | 1.4±0.2 | 1.0±0.3 | 1.0±0.4 | 0.2±0.2 | 0.5±0.2 |
| Pancreas | 52.9±0.3 | 12.5±1.0 | 0.7±0.1 | 0.4±0.1 | 0.2±0.1 | 0.2±0.0 | 0.1±0.0 |
| Muscle | 0.1±0.0 | 0.0±0.0 | 0.0±0.0 | 0.0±0.0 | 0.0±0.0 | 0.0±0.0 | 0.0±0.0 |
| Bone | 0.4±0.1 | 0.4±0.3 | 0.3±0.1 | 0.2±0.1 | 0.1±0.1 | 0.1±0.1 | 0.2±0.1 |
| Tumor | 24.1±2.1 | 45.4±3.9 | 19.9±2.0 | 9.9±0.3 | 5.6±1.2 | 4.9±1.6 | 4.3±0.9 |

**Table S2:** Biodistribution data of [^47^Sc]Sc-LF1 in T47D xenografts.

| **Organ** | **1 h** | **4 h** | **24 h** | **48 h** | **72 h** | **4 h blocked** |
| --- | --- | --- | --- | --- | --- | --- |
| Blood | 0.6±0.1 | 0.1±0.0 | 0.0±0.0 | 0.0±0.0 | 0.0±0.0 | 0.1±0.0 |
| Heart | 0.2±0.0 | 0.1±0.0 | 0.0±0.0 | 0.0±0.0 | 0.0±0.0 | 0.1±0.0 |
| Liver | 0.6±0.3 | 0.5±0.1 | 0.4±0.0 | 0.1±0.1 | 0.3±0.1 | 3.7±1.0 |
| Spleen | 0.6±0.6 | 0.2±0.1 | 0.2±0.1 | 0.2±0.0 | 0.2±0.1 | 1.7±0.3 |
| Lung | 0.5±0.5 | 0.1±0.0 | 0.1±0.0 | 0.1±0.0 | 0.0±0.0 | 1.8±0.5 |
| Kidneys | 4.0±0.4 | 2.1±0.3 | 2.0±0.2 | 0.9±0.1 | 0.9±0.3 | 2.7±1.2 |
| Stomach | 2.8±1.0 | 1.0±0.4 | 0.1±0.0 | 0.1±0.1 | 0.1±0.1 | 0.2±0.1 |
| Intestines | 1.7±1.0 | 0.5±0.3 | 0.1±0.0 | 0.1±0.0 | 0.0±0.0 | 0.1±0.0 |
| Adrenal | 2.2±1.4 | 0.8±0.5 | 0.9±0.1 | 1.2±0.1 | 0.0±0.0 | 0.2±0.1 |
| Pancreas | 49.9±5.8 | 4.7±1.5 | 0.7±0.1 | 0.5±0.1 | 0.2±0.1 | 0.2±0.1 |
| Muscle | 0.2±0.1 | 0.1±0.1 | 0.0±0.0 | 0.0±0.0 | 0.0±0.0 | 0.0±0.0 |
| Bone | 0.2±0.1 | 0.2±0.1 | 0.1±0.1 | 0.3±0.1 | 0.3±0.0 | 0.0±0.0 |
| Tumor | 9.5±2.5 | 6.1±2.4 | 3.6±1.3 | 2.4±1.9 | 0.7±0.1 | 0.0±0.0 |

**Morphological assessment**

**Table S3:** Microscopic tissue examination for kidney and pancreas of control groups and treated group of mono therapy and combination therapy

|  | **Kidney** | **Pancreas** |
| --- | --- | --- |
| **PBS** | No obvious lesions | No obvious lesions |
| **^nat^Sc-LF1 200 pmol (x6)** | No obvious lesions | No obvious lesions |
| **[^47^Sc]Sc-LF1 200 pmol (x6)** | No obvious lesions | No obvious lesions |
| **[^47^Sc]Sc-LF1 400 pmol (x6)** | No obvious lesions | No obvious lesions |
| **Everolimus 5mg/kg (x3)** | Vacuolar degeneration of some tubular epithelial cells. No other obvious lesions | No obvious lesions. |
| **[^47^Sc]Sc-LF1 400 pmol (x3)** | No obvious lesions | No obvious lesions |
| **Everolimus 5mg/kg (x3) and [^47^Sc]Sc-LF1 400 pmol (x3)** | Vacuolar degeneration of some tubular epithelial cells. No other obvious lesions | No obvious lesions |

**Table S4:** Comparison of pharmacokinetics and tumor uptake between [^47^Sc]Sc-LF1 and other GRPR antagonists

| **Tracer** | **Tumor uptake at 1 and 24h**  **(%IA/g)** | **Pancreas uptake at 1 and 24h**  **(%IA/g)** | **Kidney uptake at 1 and 24h**  **(%IA/g)** | **Liver uptake at 1 and 24h**  **(%IA/g)** | **Mice strain, sex** | **Reference** |
| --- | --- | --- | --- | --- | --- | --- |
| [^47^Sc]Sc-LF1  Prostate tumor model | 24.1 and 19.9  (45.4, 4h)  (t_1/2_=24.4h) | 52.9 and 0.7  (t_1/2_=1.5h) | 5.4 and 1.9 | 1.6 and 1.5 | Male Athymic Balb/C nude | Present  Study |
| [^47^Sc]Sc-LF1  Breast tumor model | 9.5 and 3.6  (t_1/2_=3.2h) | 49.9 and 0.7  (t_1/2_=0.9h) | 4.0 and 2.0 | 0.6 and 0.4 | Female Athymic Balb/C nude | Present  Study |
| [^177^Lu]Lu-LF1  Prostate tumor model | 42 and 18  (t_1/2_=21.8h) | 70 and 0.9  (t_1/2_=1.4h) | 7 and 1.7 | 0.7 and 0.2 | Male Athymic Balb/C nude | Kumar N et al, ACS Pharmacol. Transl. Sci, 2025 |
| [^177^Lu]Lu-NeoB  Prostate tumor model | 9 and 8  (t_1/2_=50h) | 30 and 4  (t_1/2_=7.2h) | 3.4 and 0.1 | 2.7 and 0.1 | Male NMRI-Foxn1 nu/nu | Damiana T et al, EJNMMI, 2023 |
| [^177^Lu]Lu-AMTG  Prostate tumor model | 14 and 11 | 24 and 0.5 | 4 and 1.2 | 0.7 and 0.1 | Female CB17-SCID | Holzleitner N et al, JNM, 2024 |
| [^177^Lu]Lu-RM2  Prostate tumor model | 12 and 8  (t_1/2_=40h) | 15 and 0.5  (t_1/2_=1.1h) | 3.7 and 1.8 | 1 and 0.5 | Female CB17-SCID | Holzleitner N et al, JNM, 2024 |

**Table S5:** Comparison of various parameters between [^177^Lu]Lu and [^47^Sc]Sc

| **Parameter** | **[^177^Lu]Lu** | **[^47^Sc]Sc** | **Comment** |
| --- | --- | --- | --- |
| **Theranostic pair availability** | No, [^68^Ga]Ga (mismatched pharmacokinetics) | Yes, [^44^Sc]Sc  (matched pharmacokinetics) | [^47^Sc]Sc fits true theranostic paradigm |
| **Tissue Penetration** | Moderate (up to 2 mm) | Short–moderate (0.2–0.6 mm) | [^47^Sc]Sc is safer for small tumors near critical organs |
| **Main γ emission** | 208 keV (11%) | 159 keV (68–70%) | [^47^Sc]Sc provides higher-intensity γ emission for imaging |
| **Image quality** | Moderate | Good | [^47^Sc]Sc yields better image contrast and count statistics |
| **Quantification accuracy** | Limited | Improved | Higher photon yield improves quantification with [^47^Sc]Sc |
| **Photon abundance** | Low | High | [^47^Sc]Sc offers better counting efficiency for SPECT |
| **Background signal** | Higher due to bremsstrahlung and scatter | Lower | [^47^Sc]Sc has cleaner photon spectrum for SPECT |
| **Dosimetry utility** | Limited; low counts complicate quantification | Suitable for post-therapy dosimetry | [^47^Sc]Sc enables improved therapy monitoring |
| **Dosimetry Accuracy** | Variable | High (due to matched pair) | More reliable dose planning with scandium |
